# Activation of Protein Kinase G After Repeated Cocaine Administration Is Necessary for the Phosphorylation of α-Amino-3-Hydroxy-5-Methyl-4-Isoxazolepropionic Acid Receptor GluA1 at Serine 831 in the Rat Nucleus Accumbens

**DOI:** 10.3389/fnmol.2018.00263

**Published:** 2018-07-30

**Authors:** Ju Hwan Yang, Su Yeon Seo, Jeong Hwan Oh, In Soo Ryu, Jieun Kim, Dong Kun Lee, Yeonhee Ryu, Eun Sang Choe

**Affiliations:** ^1^Department of Biological Sciences, Pusan National University, Busan, South Korea; ^2^Korea Institute of Oriental Medicine, Daejeon, South Korea; ^3^Institute of Fisheries Sciences, Pukyong National University, Busan, South Korea; ^4^Substance Abuse Pharmacology Group, Korea Institute of Toxicology, Daejeon, South Korea; ^5^Department of Physiology, School of Medicine and Institution of Health Sciences, Gyeongsang National University, Jinju, South Korea

**Keywords:** NAc, glutamate receptor, protein kinase, psychostimulant, locomotor activity

## Abstract

Phosphorylation of α-amino-3-hydroxy-5-methyl-4-isoxazolepropionic acid (AMPA) receptors in the striatum plays a crucial role in regulating the receptor-coupled signaling cascades leading to behavioral changes associated with psychostimulant exposure. The present study determined if activation of protein kinase G (PKG) contributes to the phosphorylation of AMPA receptor GluA1 subunit at the position of serine 831 (GluA1-S831) in the rat nucleus accumbens (NAc) after repeated cocaine administration. The results demonstrated that repeated intraperitoneal (i.p.) injections of cocaine (20 mg/kg) once a day for seven consecutive days significantly increased the level of phosphorylated (p)GluA1-S831. This increase was decreased by the intra-NAc infusion of either the cyclic guanosine monophosphate (cGMP) analog, Rp-8-Br-PET-cGMPS (5 nmol/1 μL), or the PKG inhibitor, KT5823 (2 nmol/1 μL). Repeated cocaine administration increased PKG binding activity to GluA1. This increase in GluA1-S831 phosphorylation after repeated cocaine administration was decreased by the intra-NAc infusion of the synthetic peptide (Tat-tagged interfering peptide (Tat-GluA1-i)), that interferes with the binding of PKG to GluA1. Intra-NAc infusion of the interfering peptide also reduced the repeated cocaine-induced increase in locomotor activity. These findings suggest that activated PKG, after repeated exposure to cocaine, binds to AMPA receptor GluA1 and is required for the phosphorylation of S831, contributing to behavioral changes.

## Introduction

The α-amino-3-hydroxy-5-methyl-4-isoxazolepropionic acid (AMPA) receptor is an ionotropic glutamate receptor that plays a crucial role in drug-mediated synaptic plasticity by regulating fast ion conductance (Bowers et al., 2010; Oh et al., 2013; Hanley, 2014). The GluA1-4 subunits consisting of AMPA receptor have structural homology in their domain organization: amino-terminus, ligand binding, channel pore-forming transmembrane and carboxyl-terminus (CT) (Nakagawa, 2010; Shanks et al., 2010). The CT of the GluA1 subunit contains two major phosphorylation sites, serine residue 831 (S831) for protein kinase C (PKC) and Ca^2+^-calmodulin-dependent protein kinase II (CaMKII), and serine 845 (S845) for protein kinase A (PKA) and protein kinase G (PKG) (Serulle et al., 2007; Hanley, 2014). Phosphorylated S831 and S845 of GluA1 are known to be involved in the regulation of AMPA receptor functions separately or together. For instance, phosphorylation of S845 regulates open probability and open duration of AMPA receptors via PKA activation coupled to dopamine D1 receptors (Banke et al., 2000; Han and Whelan, 2009), while phosphorylation of S831 regulates AMPA conductance via CaMKII or PKC activation coupled with glutamate receptors (Derkach et al., 1999; Jenkins and Traynelis, 2012). In addition to single phosphorylation of the receptors, dual phosphorylation of S831 and S845 regulates membrane trafficking of AMPA receptors and synaptic insertion (Gao et al., 2006; Derkach et al., 2007). These findings suggest that phosphorylation of the serine residue contributes to the alternation of AMPA receptor functions.

Previous studies showed that psychostimulants, such as cocaine, upregulate AMPA receptor functions through GluA1 subunit at the position of serine 831 (GluA1-S831) phosphorylation in the rat striatum. For instance, repeated exposure to cocaine increases phosphorylation of GluA1-S831 in the nucleus accumbens (NAc) (Schierberl et al., 2011). Phosphorylation of GluA1-S845 increases cocaine-induced conditioned place preference scores via AMPA receptors trafficking in the NAc core (Zheng et al., 2015). Repeated exposure to amphetamine increases S845 and S831 phosphorylation, and PKA and CaMKII expression in the postsynaptic density of the rat NAc (Wang et al., 2017). These findings suggest that GluA1 phosphorylation at different serine residues by protein kinases in the NAc is involved in drug dependance.

It is known that S831 is phosphorylated by PKC and CaMKII, while S845 is phosphorylated by PKA in transfected cells expressing GluA1 and PKG in the hippocampus and hippocampal primary cultured neurons (Roche et al., 1996; Mammen et al., 1997; Serulle et al., 2007). These findings suggest that phosphorylation sites on the two serine residues in GluA1 subunit are associated with glutamate and/or dopamine receptor-coupled protein kinase activity after cocaine administration. For instance, repeated cocaine administration phosphorylates GluA1-S831 in the dorsal striatum by glutamate receptor-linked PKC activation (Kim et al., 2009), while acute cocaine administration upregulates GluA1-S845 phosphorylation in the mouse neostriatum via dopamine D1 receptor-coupled PKA activation (Snyder et al., 2000).

Repeated exposure to cocaine upregulates the nitric oxide (NO)/cyclic guanosine monophosphate (cGMP)/PKG signaling cascades in the rat dorsal striatum in a Ca^2+^-dependent manner (Lee et al., 2010, 2013; West and Tseng, 2011). Previously, we demonstrated that activation of PKG after repeated cocaine administration upregulates AMPA receptor GluA1-S845 phosphorylation in the rat NAc (Seo et al., 2013). These findings suggest that AMPA receptor GluA1 subunit can be a common substrate for a group of Ca^2+^-dependent protein kinases in the striatum after repeated exposure to cocaine, contributing to behavioral changes. Previous studies show that phosphorylation of AMPA receptors in the brain regions, related to reward after psychostimulant exposure, causes behavioral changes (Boudreau and Wolf, 2005; Ferrario et al., 2010; Yamamoto and Zahniser, 2012; Seo et al., 2013). However, mechanisms underlying phosphorylation of the serine residues in AMPA receptor GluA1 subunit by protein kinases associated with drug exposure remain to be identified. In this study, we demonstrated that activation of PKG is necessary for GluA1-S831 phosphorylation in the NAc after repeated cocaine administration and that the interaction of PKG and GluA1 regulates behavioral changes.

## Materials and Methods

### Animals

Adult male Sprague-Dawley rats (7 weeks, 250–300 g) were obtained from Hyo-Chang Science Co. (Daegu, South Korea). The rats were allowed to acclimate for a minimum of 3 days, housed as a pair in a controlled environment, and maintained on a 12 h light/dark cycle during all experimental treatments. In addition, temperature and humidity were maintained at 21–23°C and 45%–55%, respectively. Food and water were provided *ad libitum*. On the day of the experiment, injections were given in a quiet room to minimize stress to the animals. All animal-use were approved by the Institutional Animal Care and Use Committee and were carried out in accordance with the provisions of the NIH Guide for the Care and Use of Laboratory Animals.

### Drugs

**Figure 1:**
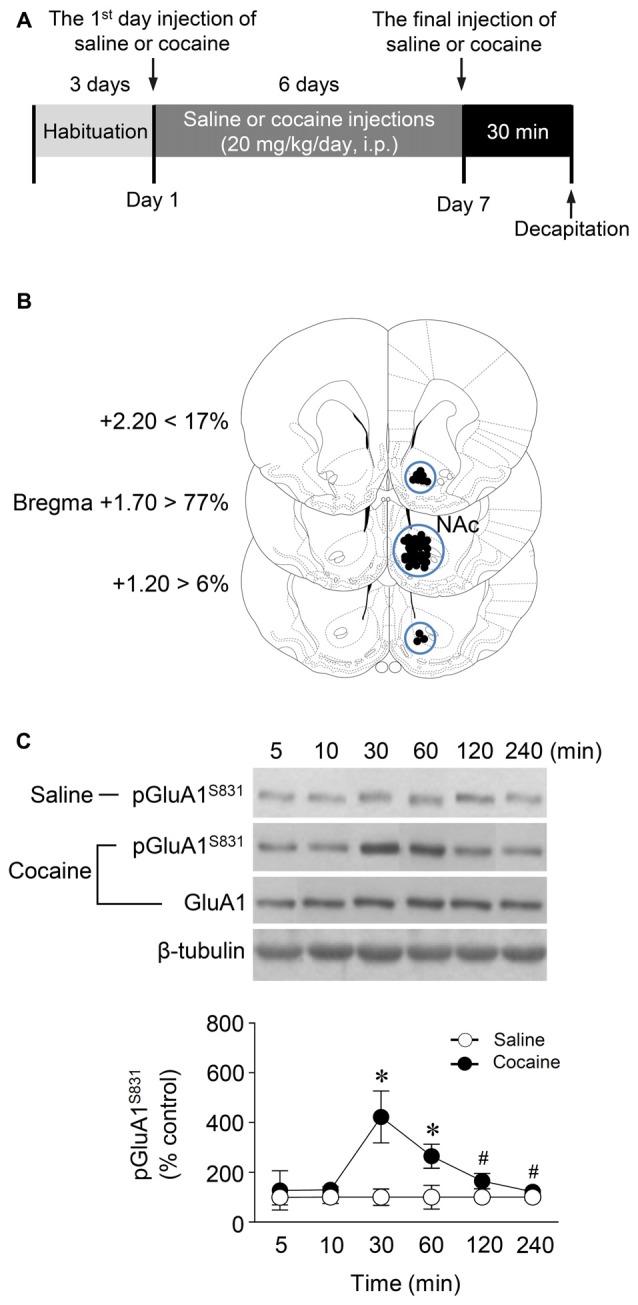
Phosphorylation of GluA1-S831 after repeated cocaine administration in the Nucleus accumbens (NAc). **(A)** Timeline for western immunoblotting analysis. **(B)** Brain sections showing statistics for area punched out for western immunoblotting analysis (blue circles). **(C)** GluA1-S831 phosphorylation was significantly increased at 30 min after repeated cocaine administration and sustained up to 60 min as compared with the saline control group. This increase was returned to the basal level at the 120 min time point (Interaction, *F*_(5,30)_ = 16.54, *df* = 5, *p* < 0.05; Time, *F*_(5,30)_ = 16.54, *df* = 5, *n* = 4 rats per group; *p* < 0.05; two-way repeated measures analysis of variance (RM ANOVA) followed by Bonferroni’s multiple comparisons test. *p < 0.05 vs. repeated saline group; ^#^*p* < 0.05 vs. repeated cocaine group at the 30 min time point. Western immunoblotting was performed repeatedly four times.

All pharmacological drugs, except for cocaine (Balgopia, Louvain-La-Neuve, Belgium), were purchased from Tocris Bioscience (Bristol, UK). The cGMP analog, Rp-8-Br-PET-cGMPS (5 nmol), and the PKG inhibitor, KT5823 (2 nmol), were dissolved in the mixture of artificial cerebro-spinal fluid (aCSF) containing (mM) 123 NaCl, 0.86 CaCl_2_, 3.0 KCl, 0.89 MgCl_2_, 0.50 NaH_2_PO_4_, and 0.25 Na_2_HPO_4_ aerated with 95% O_2_/5% CO_2_, pH 7.2–7.4 and 99% dimethylsulfoxide (DMSO). Rp-8-Br-PET-cGMPS and KT5823 were dissolved in the solvent in the ratio of 8:2 and 5:5, respectively. Concentrations of the drugs were determined by our previous study (Seo et al., 2013). The same DMSO-aCSF mixture solution was used as the vehicle control for a given drug. All drugs were freshly prepared on the day of the experiment and adjusted to a pH of 7.2–7.4, if necessary. The rats received repeated intraperitoneal (i.p.) injections of saline (0.9% sodium chloride, 1 mL/kg) or cocaine (20 mg/kg) once a day for seven consecutive days (Figure 1A). Cocaine was injected in a volume of 1 mL/kg in physiological saline.

### Western Immunoblotting

Western immunoblotting analysis was performed as previously described (Choe et al., 2004). Rats were deeply anesthetized with 8% chloral hydrate (5.8 mL/kg, i.p.). Brains were decapitated 30 min after the final injection of saline or cocaine except for a time point study in which they were removed at six different time points. The brains were removed, frozen in isopentane at −70°C, and stored in a deep freezer until use. The sections were serially cut using a cryostat (Leica biosystems, Nussloch, Germany) at −25°C, after which the injected right NAc was removed with a steel borer (2 mm inner diameter; Figure 1B). Tissues were then transferred to homogenization buffer containing 10 mM Tris-HCl, pH 7.4, 5 mM NaF, 1 mM Na_3_VO_4_, 1 mM EDTA and 1 mM EGTA, sonicated three times for 9 s each and then incubated on ice for 1 h. After sonicating, samples were centrifuged at 13,000 rpm for 30 min at 4°C. The pellet, which primarily contains nuclei and large debris, was discarded, and the supernatant was centrifuged again at 13,000 rpm for 30 min at 4°C. The concentration of the solubilized proteins in the supernatant fraction was determined based on the Bradford protein assay using a Bio-Rad Protein Assay (Bio-Rad Laboratories, Hercules, CA, USA). The proteins in the supernatant were resolved using 10% bisacrylamide gel electrophoresis, after which the separated proteins were transferred to a nitrocellulose membrane. The membrane was blocked with blocking buffer containing 5% skim milk in a mixture of Tris-buffered saline and 0.1% Tween-20 (TBST), and washed three times for 10 min each with TBST. After washing, the membrane was probed with a rabbit primary antiserum against phosphorylated (p)GluA1-S831 (1:1,000; Cell Signaling Technology, Danvers, MA, USA) for 18 h at 4°C on a shaker. The membrane was washed again and incubated with peroxidase-labeled goat anti-rabbit IgG (1:1,000; KPL, Gaithersburg, MD, USA) under the same conditions as the primary antiserum for 1 h at room temperature. Next, immunoreactive protein bands were detected on X-ray films using Westsave UP (Ab Frontier, Seoul, South Korea, Ratio of reagents A to B = 1:500). The same membrane was also probed for β-tubulin (1:2,000; Santa Cruz Biotechnology, Santa Cruz, CA, USA) to normalize the blots. Rabbit primary antiserum against unphosphorylated GluA1 (Cell Signaling Technology) was diluted to 1:1,000 in 2% skim milk in TBST. Immunoreactive protein bands visualized on films were semi-quantified using an imaging digital camera and the NIH Image 1.62 software as previously described (Ahn and Choe, 2009; Kim et al., 2009).

### Surgery and Intra-NAc Infusion of Drugs

**Figure 2:**
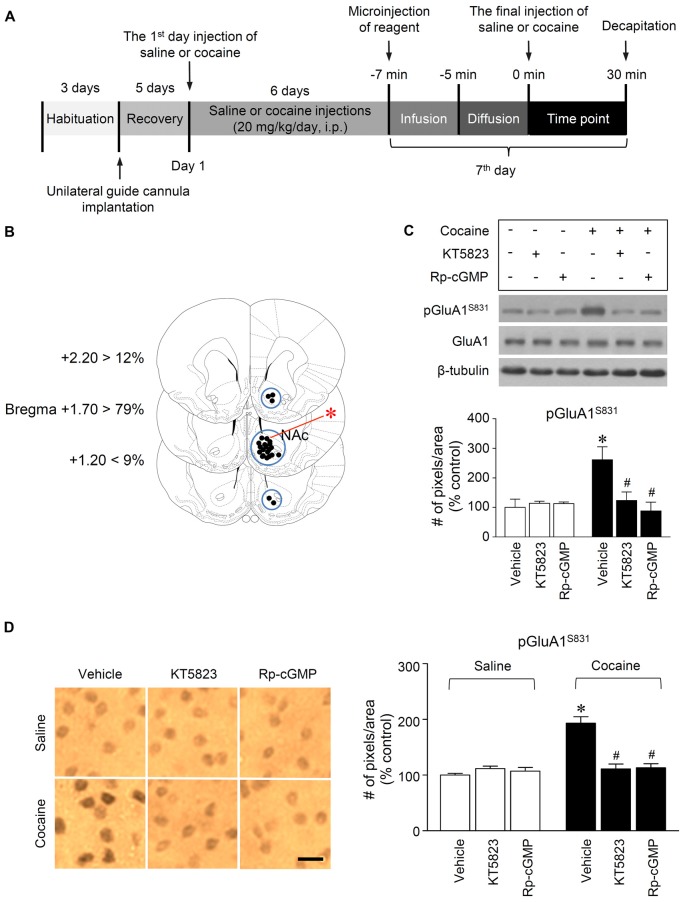
PKG-regulated phosphorylation of GluA1-S831 after repeated cocaine administration in the NAc. **(A)** Timeline for western immunoblotting and immunohistochemical analyses. **(B)** Brain sections showing statistics for the reconstruction of microinjections of drugs, and areas punched out for western immunoblotting analysis (blue circles). **(C,D)** Intra-NAc infusion of the PKG inhibitor, KT5823 (2 nmol), and the cGMP analog, Rp-8-Br-PET-cGMPS (5 nmol), significantly decreased the repeated cocaine-induced increase in GluA1-S831 phosphorylation in western immunoblotting (Treatment, *F*_(5,18)_ = 21.5; *df* = 23; *n* = 4 rats per group; *p* < 0.05; *p < 0.05 vs. vehicle + repeated saline group; ^#^*p* < 0.05 vs. vehicle + repeated cocaine group; one-way ANOVA followed by Tukey’s multiple comparisons test) and immunohistochemical (Treatment, *F*_(5,18)_ = 21.62; *df* = 23; *n* = 4 rats per group; *p* < 0.05; *p < 0.05 vs. vehicle + repeated saline group; ^#^*p* < 0.05 vs. vehicle + repeated cocaine group; one-way ANOVA followed by Tukey’s multiple comparisons test) analyses. Photomicrographs were taken from in the border of the core and shell as shown in **(B)** (red asterisk). Western immunoblotting and immunohistochemistry were performed repeatedly four times.

**Figure 3:**
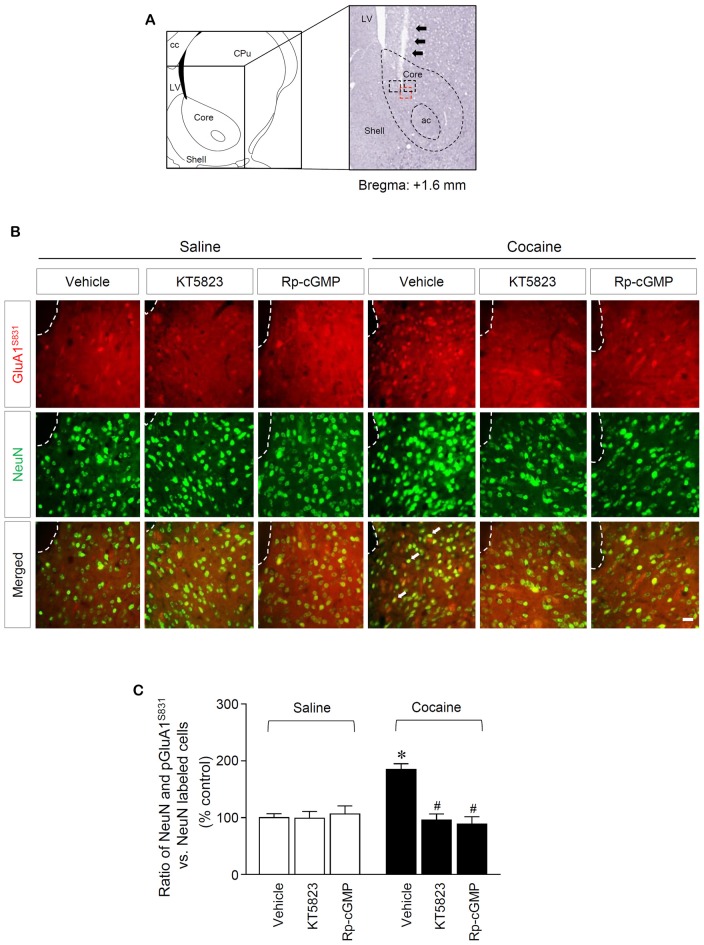
PKG-regulated co-expression of GluA1-S831 with NeuN after repeated cocaine administration in the NAc. **(A)** A schematic diagram showing a Nissl-stained brain section from the Bregma +1.6 mm represented an injection needle tract (arrows) in the core of the NAc. **(B)** Double-immunofluorescent staining showed that repeated cocaine administration significantly decreased the ratio of pGluA1-S831 and NeuN co-localization after the intra-NAc infusion of KT5823 (2 nmol) or Rp-8-Br-PET-cGMPS (5 nmol). The dotted lines and white arrows represent injection tracks and pGluA1-S831 and NeuN double-stained cells, respectively. A bar represents 500 μm. ac, anterior commissure; cc, corpus callosum; CPu, caudate putamen; LV, lateral ventricle. **(C)** The ratio of NeuN and pGluA1-S831 double labeled vs. NeuN-labeled cells in co-immunofluorescent analysis (Treatment, *F*_(5,36)_ = 71.28; *df* = 41; *n* = 7 rats per group; *p* < 0.05; *p < 0.05 vs. vehicle + repeated saline group; ^#^*p* < 0.05 vs. vehicle + repeated cocaine group; one-way ANOVA followed by Tukey’s multiple comparisons test).

Rats were anesthetized with 8% chloral hydrate, and then placed in a stereotaxic apparatus. Under aseptic conditions, a 23-gauge stainless steel guide cannula (0.29 mm inner diameter, 10 mm in length) was implanted into the right hemisphere of the NAc (1.6 mm anterior to the bregma, 1.2 mm right of the midline and 7.5 mm below the surface of the skull). The guide cannula was sealed with a stainless steel wire of the same length. The rats were then allowed to recover from surgery for at least 5 days prior to the experiment. On the day of the experiment, the inner steel wire was replaced with a 30-gauge stainless steel injection cannula (0.15 mm inner diameter, 12.5 mm in length) protruding 2.5 mm below the tip of the guide cannula. Throughout the experiments, all drugs were infused unilaterally into the central part of the right NAc 5 min prior to the final injection of cocaine or saline in a volume of 1 μL at a rate of 0.5 μL/min in freely moving rats (Figure 2A). The progress of the injection was monitored by observing the movement of a small air bubble along the length of pre-calibrated PE-10 tubing, which is connected between the injection cannula and a 2.5 μL Hamilton microsyringe (Thermo Fisher Scientific, Pittsburgh, PA, USA). After completing the injection, the injector was left in place for an additional 5 min to reduce any possible backflow of the solution along the injection tract. The physical accuracy of the injection was verified by the reconstruction of microinjection placements (Figure 2B). The possibility of gliosis caused by the implantation of the guide cannula and the infusion of drugs dissolved in DMSO/aCSF was verified by Nissl staining (Figure 3A).

### Immunohistochemistry

Immunohistochemistry was performed as previously described (Choe et al., 2004). Perfused brains were serially cut in 30 μm sections using a sliding microtome on a freezing plate. Three sections per brain were collected and processed for immunohistochemistry. The sections were washed with 1% Triton X-100 in phosphate buffered saline (PBS, pH 7.2) for 10 min. The sections were then pre-incubated in 0.6% H_2_O_2_ in the dark for 30 min after being washed three times in PBS for 10 min. The sections were incubated with normal goat serum (Vector Laboratories, Burlingame, CA, USA) to eliminate the possibility of non-specific cross reactions. Finally, the sections were incubated with pGluA1-S831 antiserum (Santa Cruz Biotechnology) at 1:500 for 20 h at 4°C on a rolling shaker. After washing the sections three times with PBS for 10 min, the sections were incubated in biotinylated-goat anti-rabbit IgG (1:1,000; Vector Laboratories) for 1 h, and in avidin–biotin–peroxidase reagents (Vector Laboratories) for 1 h at room temperature on a rolling shaker. Diaminobenzidine was used as the chromogen and NiCl_2_ was added to enhance the reaction product. Finally, the sections were mounted onto gelatin-coated slides. Expression levels of pGluA1-S831 near the injection tract in the NAc were semi-quantified using the NIH Image 1.62 software as previously described (Ahn and Choe, 2009; Kim et al., 2009).

### Double-Immunofluorescence

Rats were deeply anesthetized with a mixture of Zoletil 50 (75 with a mixture of Zoletil 50 μL/kg; Virbac Korea, Seoul, South Korea) and Rompun (50 μL/kg; Bayer Korea, Seoul, Korea) via i.p. injections and then transcardially perfused with 4% paraformaldehyde in PBS at 4°C. The brains were then removed and post-fixed in a solution of 10% sucrose in 4% paraformaldehyde for 2 h at 4°C, after which they were placed in 20% sucrose in PBS and held at 4°C overnight (Choe et al., 2004). Using a freezing sliding microtome, 16 μm frozen sections were obtained. Three sections per brain were used for the double-immunofluorescence staining. The sections were blocked with a blocking buffer containing 4% normal goat serum and 1% bovine serum albumin in PBS, then washed with PBS three times for 10 min each. The sections were then incubated in a mixture of rabbit or mouse antiserum against pGluA1-S831 (Santa Cruz Biotechnology) and the neuron-specific nuclear protein (NeuN; Abcam, Cambridge, MA, USA) at 1:50 and 1:2,000, respectively, for 24 h at 4°C on a shaker. After rinsing the sections several times in PBS, the sections were incubated in a mixture of goat anti-mouse and rabbit secondary antisera conjugated to Alexa Fluor 488 and 594 (1:1,000; Abcam) for 2 h. The sections were rinsed three times and mounted on slide glasses, cover-slipped, and monitored using a fluorescence camera (Carl Zeiss Microscopy, Jena, Germany) with specific filters for detecting specific wavelengths. Images of the sections were obtained by using ZEN 2012 SP2 (blue edition) image-processing software. The photographed area in each brain section was shown in red square and double-immunofluorescent labeled cells were counted in red and black squares (29.16 mm^2^) which represent the end of an injector (Figure 3A). Quantification of pGluA1-S831 immunoreactivity was performed by calculating the ratio of NeuN and pGluA1-S831 double stained cells to total NeuN-stained cells. The values of each group were then compared.

### Co-immunoprecipitation (Co-IP) Assay

Rats were deeply anesthetized with 8% chloral hydrate and decapitated 30 min after the final injection of saline or cocaine. The brains were quickly removed, frozen in isopentane at −70°C, and stored in a deep freezer. Tissue samples from the NAc of 4~5 rats were pooled and homogenized on ice in binding buffer containing 50 mM Tris-HCl (pH 7.5), 150 mM NaCl, 10% glycerol, 1% Triton X-100, 1% Igepal CA-630, 1% deoxycholate, 2 mM EDTA, 1 mM phenylmethylsulfonyl fluoride and protease and phosphatase inhibitor cocktail (Thermo Scientific, Rockford, IL, USA) and put on ice for 30 min. Homogenates were centrifuged at 10,000 *g* for 20 min at 4°C. The concentration of solubilized proteins in the supernatant was determined using a Bio-Rad protein assay kit (Bio-Rad Laboratories) based on the Bradford protein assay. Solubilized proteins (500 μg) were incubated with a rabbit antiserum against GluA1 (Cell Signaling Technology) overnight at 4°C. The complex was precipitated with 50% protein G agarose/sepharose bead slurry (GE Healthcare Bio-Sciences, Piscataway, NJ, USA) for 5 h with gentle shaking at 4°C. After incubating for 5 h, the samples were centrifuged at 1000 *g* for 5 min at 4°C. Pellets were washed with binding buffer five times and all supernatants were removed carefully without touching the pellets. 2× SDS loading buffer was added to the pellets which were then boiled for 5 min at 95°C. Beads were spun down and the supernatant was collected without touching the beads and kept at −70°C until use. Proteins were separated on 10% bisacrylamide gels and transferred to nitrocellulose membranes. The membranes were incubated with rabbit antiserum against pGluA1-S831 (1:1,000, EMD Millipore, Billerica, MA, USA), GluA1 (1:1,000, EMD Millipore) or PKG (1:1,000, Abcam). After incubating in peroxidase-labeled goat anti-rabbit IgG (1:1,000; KPL), membranes containing immunoreactive protein bands were developed on X-ray films using Westsave UP (Ab Frontier). The visualized immunoreactive protein bands in the films were semi-quantified as previously described (Ahn and Choe, 2009; Kim et al., 2009).

### Synthesis and Intra-NAc Infusion of Peptide

Cell permeable peptide was synthesized to interfere with the binding of PKG to AMPA receptor GluA1 in the NAc (Peptron, Daejeon, South Korea). The peptide contains a PKG binding domain (amino acids 850–873) on GluA1 subunit CT domain (Figure 5A) (Serulle et al., 2007). As a control of interfering peptide, scrambled peptide was also designed to determine sequence-specific interaction of PKG with GluA1. To enhance cell-permeability of the peptides, HIV-1 Tat (YGRKKRRQRRR) sequence was conjugated to its N-terminus of either interfering (Tat-tagged interfering peptide (Tat-GluA1-i)) or scrambled (Tat-tagged scrambled peptide (Tat-GluA1-c)) peptide. The peptides were then dissolved in aCSF and their concentrations were adjusted to 0.2 and 2 nmol. Like drug infusion, peptides were infused unilaterally into the central part of the right NAc 7 min prior to the final injection of saline or cocaine in a volume of 1 μL at a rate of 0.5 μL/min in freely moving rats. After completing the injection, the injector was left in place for an additional 5 min to reduce any possible backflow of the peptide solution along the injection tract.

**Figure 4:**
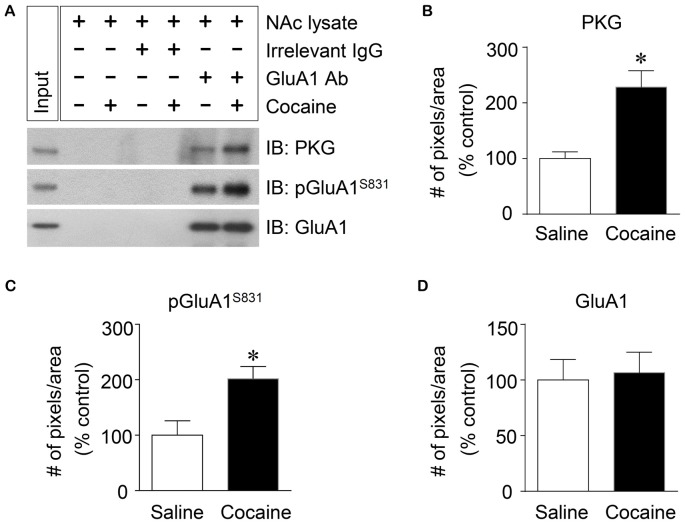
Elevation of PKG binding activity to GluA1-CT after repeated cocaine administration in the NAc. **(A)** Co-IP assay demonstrated that PKG and GluA1 binding was significantly increased after repeated cocaine administration. **(B)** Quantification of PKG immunoreactivity bound to GluA1 (*t* = 9.051, *df* = 8, *n* = 5 tissue samples per group, *p* < 0.05; *p < 0.05 vs. repeated saline group; unpaired *t*-test). **(C)** Quantification of pGluA1-S831 immunoreactivity in the GluA1 precipitates (*t* = 6.738, *df* = 8, *n* = 5 tissue samples per group, *p* < 0.05; *p < 0.05 vs. repeated saline group; unpaired *t*-test). **(D)** Quantification of GluA1 immunoreactivity in the GluA1 precipitates (*t* = 0.5769, *df* = 8, *n* = 5 tissue samples per group; *p* = 0.5799; *p < 0.05 vs. repeated saline group; unpaired *t*-test). Co-IP assay was performed repeatedly three times.

**Figure 5:**
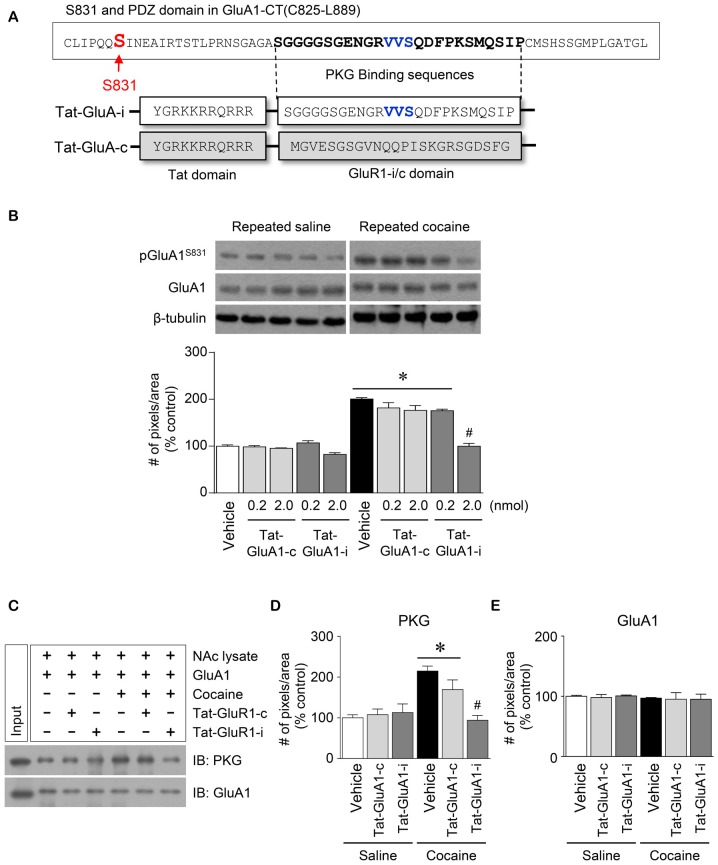
Regulation of GluA1-S831 phosphorylation by PKG binding to GluA1-CT domain in the NAc after repeated cocaine administration. **(A)** Tat-GluA1-i was synthesized to interfere PKG binding to the binding motif, while Tat-GluA1-c for a control. **(B)** Intra-NAc infusion of Tat-GluA1-i (2 nmol) significantly decreased the repeated cocaine-induced increase in GluA1-S831 phosphorylation, however Tat-GluA1-c did not (Treatment, *F*_(9,40)_ = 62.11; *df* = 49; *n* = 5 rats per group; *p < 0.05 vs. vehicle + repeated saline vehicle group; ^#^*p* < 0.05 vs. vehicle + repeated cocaine vehicle group; one-way ANOVA followed by Tukey’s multiple comparisons test). **(C)** Co-IP analysis showed that intra-NAc infusion of Tat-GluA1-i (2 nmol) significantly decreased PKG binding to GluA1 which was increased by repeated cocaine administration. **(D)** Quantification of PKG immunoreactivity bound to GluA1 (Treatment, *F*_(5,12)_ = 27.08; *df* = 17; *n* = 3 tissue samples per group; *p* < 0.05; *p < 0.05 vs. vehicle + repeated saline vehicle group; ^#^*p* < 0.05 vs. vehicle + repeated cocaine vehicle group; one-way ANOVA followed by Tukey’s multiple comparisons test). **(E)** Quantification of GluA1 immunoreactivity in the GluA1 precipitates (Treatment, *F*_(5,12)_ = 0.4616; *df* = 17; *n* = 3 tissue samples per group; *p* = 0.7975; one-way ANOVA followed by Tukey’s multiple comparisons test). Western immunoblotting and Co-IP assay were performed repeatedly three times.

### Behavioral Assessments

**Figure 6:**
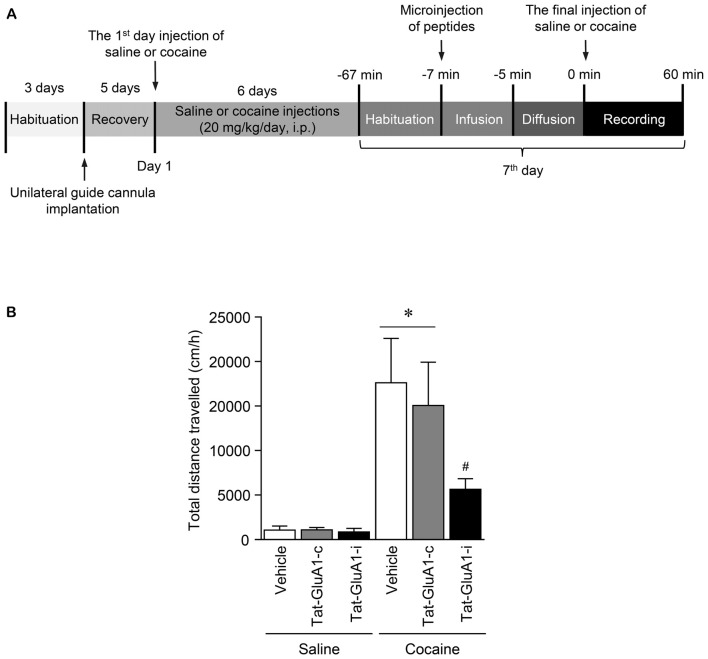
Regulation of locomotor activity by PKG binding to GluA1-CT domain in the NAc after repeated cocaine administration. **(A)** Timeline for locomotor activity analysis. **(B)** Intra-NAc infusion of Tat-GluA1-i, but not Tat-GluA1-c, significantly attenuated the repeated cocaine-induced increase in locomotor activity (Treatment, *F*_(5,54)_ = 68.82; *df* = 59; *n* = 10 rats per group; *p* < 0.05; *p < 0.05 vs. vehicle + repeated saline group; ^#^*p* < 0.05 vs. vehicle + repeated cocaine group). One-way ANOVA followed by Tukey’s multiple comparisons test.

Behavioral assessments were conducted as previously described (Liu et al., 2006). Under sound-attenuated and illuminated conditions, locomotor activity after the final injection of drug was measured in an open field with an infrared photocell-based Opto-Varimex-4 Auto-Track (Columbus Instruments, Columbus, OH, USA). Rats were placed in the standard transparent rectangular chamber (44.5 cm × 44.5 cm) and habituated for 1 h prior to injections to avoid environmental variations of behavioral tests (Figure 6A). Three pairs of sensors were positioned at *x*, *y* (horizontal), and *z* (vertical, placed above the animal’s normal height) axes in the cage to provide coordinates of the rat moving in the locomotor testing chamber. Each pair of sensor produces 16 infrared light beams intersecting the animal cage (beam scan rate = 10 Hz). The Auto-Track system senses the presence of animals by receiving infrared beam interruptions. Locomotor activity was recorded at 10 min intervals over an hour period after the final injection of the drug and the measurements were then transferred to a computer with operating software (version 4.99B, Columbus Instruments).

### Statistics

Differences in the number of immunoreactive pixels per measured area and total distance traveled between groups were determined by unpaired *t*-test or one-way analysis of variance (ANOVA) followed by the Tukey’s multiple comparison test or two-way ANOVA with repeated measure (two-way RM ANOVA) using GraphPad Prism 6 (GraphPad Software Incorporation, San Diego, CA, USA). Sample sizes used in this study were justified by a power analysis using R-studio program (Rstudio, Boston, MA, USA). Data were expressed as the mean ± SEM for each group. A statistical significance was determined by *p* < 0.05.

## Results

### Repeated Cocaine Administration Increased GluA1-S831 Phosphorylation via PKG Activation in the NAc

This study was performed to determine whether repeated cocaine administration phosphorylates GluA1-S831 because it phosphorylates GluA1-S845 in the NAc (Seo et al., 2013). Repeated systemic injections of cocaine significantly increased pGluA1-S831 immunoreactivity in the NAc at 30 min, sustained up to 60 min and returned to basal level at 120 min when compared with the saline control group. However, repeated saline or cocaine administration did not alter the immunoreactivity of unphosphorylated GluA1 throughout the experiments (Figure 1C: Interaction, *F*_(5,30)_ = 16.54, *df* = 5, *p* < 0.05; Time, *F*_(5,30)_ = 16.54, *df* = 5, *p* < 0.05). Since repeated cocaine administration increased pGluA1-S831 level, the next experiment was conducted to determine whether PKG activation can regulate the phosphorylation of GluA1-S831 after repeated cocaine administration. Intra-NAc infusion of either the cGMP analog, Rp-8-Br-PET-cGMPS (5 nmol), or the PKG inhibitor, KT5823 (2 nmol), significantly decreased the repeated cocaine-induced increase in pGluA1-S831 immunoreactivity when compared with the repeated saline treated group in both western immunoblotting (Figure 2C: Treatment, *F*_(5,18)_ = 21.5; *df* = 23; *p* < 0.05) and immunohistochemical (Figure 2D: Treatment, *F*_(5,18)_ = 21.62; *df* = 23; *p* < 0.05) analyses. Similarly, double-immunofluorescent analysis showed that the increased number of pGluA1-S831 and NeuN labeled cells after repeated cocaine administration was significantly decreased by the intra-NAc infusion of Rp-8-Br-PET-cGMPS or KT5823 (Figures 3B,C: Treatment, *F*_(5, 36)_ = 71.28; *df* = 41; *p* < 0.05). The real values of the rates of changes in the immunoreactivity of pGluA1-S831 and the number of pGluA1-S831 and NeuN double labeled cells in the NAc are listed in Supplementary Tables S1–S3.

### Repeated Cocaine Administration Increased PKG Binding to GluA1-CT

PKG is known to bind at PDZ motif in the CT of GluA1 (Serulle et al., 2007), which is necessary for phosphorylating GluA1-S845. Since the activation of cGMP or PKG in the NAc is involved in the phosphorylation of GluA1-S831, this study was conducted to determine whether PKG binding to GluA1 has the capacity to regulate GluA1-S831 phosphorylation in the NAc using Co-IP assay. Repeated cocaine, but not repeated saline, administration significantly increased the immunoreactivities of PKG (Figures 4A,B: *t* = 9.051, *df* = 8; *p* < 0.05) and pGluA1-S831 (Figures 4A,C: *t* = 6.738, *df* = 8; *p* < 0.05). However, the immunoreactivity of GluA1 for a control did not alter after both repeated cocaine and saline administration (Figures 4A,D *df* = 8; *p* = 0.5799). The real values of the rates of changes in the immunoreactivity of PKG, pGluA1-S831 or GluA1 in the NAc are listed in Supplementary Table S4.

### Blockade of PKG Binding to PDZ Motif in GluA1-CT Reduced the Repeated Cocaine-Induced Increase in GluA1-S831 Phosphorylation

This study was conducted to determine if blockade of PKG binding to PDZ motif alters the repeated cocaine-induced increase in GluA1-S831 phosphorylation. Tat-GluA1-i was designed to interfere with the binding of PKG to PDZ motif in the GluA1-CT (Figure 5A). Intra-NAc infusion of Tat-GluA1-i (2 nmol), but not Tat-GluA1-c, significantly decreased the repeated cocaine-induced increase in pGluA1-S831 immunoreactivity (Figure 5B: Treatment, *F*_(9,40)_ = 62.11; *df* = 49; *p* < 0.05). In addition, we also conducted Co-IP assay to determine if Tat-GluA1-i peptide prevents PKG from binding to GluA1-CT. Intra-NAc infusion of Tat-GluA1-i peptide (2 nmol) significantly decreased the repeated cocaine-induced increase in PKG (Figures 5C,D: Treatment, *F*_(5,12)_ = 27.08; *df* = 17; *p* < 0.05), however, the immunoreactivity of GluA1 did not (Figures 5C,E: Treatment, *F*_(5,12)_ = 0.4616; *df* = 17; *p* = 0.7975). The real values of the rates of changes in the immunoreactivity of pGluA1-S831 (Figure 5B), and PKG and GluA1 (Figure 5C) in the NAc are listed in Supplementary Tables S5, S6.

### Blockade of PKG Binding to GluA1-CT in the NAc Decreased the Repeated Cocaine-Induced Increase in Locomotor Activity

This study was performed to determine whether the interaction of PKG and GluA1-CT in the NAc is required for changes in locomotor activity after repeated cocaine administration. Intra-NAc infusion of Tat-GluA1-i peptide (2 nmol), but not Tat-GluA1-c peptide (2 nmol), significantly decreased the repeated cocaine-induced increase in locomotor activity and sustained up to 60 min (Figure 6B: Treatment: *F*_(5,54)_ = 68.82; *df* = 59; *p* < 0.05). The real values of the rates of changes in locomotor activities after intra-NAc infusion of Tat-peptides prior to the final injection of saline or cocaine are listed in Supplementary Table S7.

## Discussion

The present findings show that repeated cocaine administration increases AMPA GluA1-S831 phosphorylation in the NAc via activation of cGMP or PKG. This increase was found to occur by increased binding of PKG to PDZ domain in the CT region of AMPA receptor GluA1 subunit. These findings suggest that there is an interaction between PKG activation and GluA1-S831 phosphorylation, leading to behavioral changes.

In the present study, inhibition of either cGMP or PKG in the NAc hinders the repeated cocaine-induced increase in the phosphorylation of GluA1 at S831. Similar interactions occur between protein kinases and GluA1 phosphorylation. Repeated cocaine administration seems to regulate S845 or S831 phosphorylation via activation of CaMKII, ERK1/2 and PKC by stimulating glutamate receptors in the dorsal striatum (Kim et al., 2009; Oh et al., 2013). In addition, intra-NAc infusion of either cGMP analog or PKG inhibitor also attenuates the repeated cocaine-induced increase in locomotor activity (Seo et al., 2013). These findings suggest that activated PKG after repeated exposure to cocaine alters behaviors by interacting with GluA1-CT. One of the interactions regulating GluA1-S831 phosphorylation would be the NO/cGMP/PKG signaling cascade. Activation of the NO/cGMP/PKG pathway after repeated exposure to cocaine in the NAc is necessary for phosphorylating GluA1-S831. Repeated cocaine administration activates PKC and PKA which are involved in the cocaine reinstatement by stimulating group I mGluRs in the dorsal striatum, and dopamine D1 receptors in the neostriatum, respectively (Snyder et al., 2000; Schmidt and Pierce, 2010; Oh et al., 2013; Schmidt et al., 2013). Activation of these kinases in turn phosphorylates *N*-methyl D-aspartate receptors and upregulates Ca^2+^ signaling cascades (Tingley et al., 1997; Impey et al., 1999; Dudman et al., 2003). The Ca^2+^-dependent kinases linked to the receptor signaling cascades and/or PKC and PKA then phosphorylate the downstream effector, neuronal NO synthase, upregulating NO production in the rat dorsal striatum (Nakane et al., 1991; Hayashi et al., 1999; Lee et al., 2010). An increase in NO efflux is found to activate cGMP and/or PKG in the rat dorsal striatum (Domek-Łopacińska and Strosznajder, 2005). Taken together, these findings suggest that activation of the NO/cGMP/PKG signaling cascades in the NAc after repeated cocaine administration can regulate AMPA receptors by phosphorylating GluA1-S831. This notion would be supported by the finding that activation of PKG after repeated cocaine administration increases GluA1-S845 phosphorylation in the rat NAc (Seo et al., 2013) even though kinetics underlying phosphorylation of the two serine residues in response to repeated cocaine administration remain to be determined.

The present finding shows that binding of PKG to AMPA GluA1-CT is increased after repeated cocaine administration in the NAc, suggesting that direct interaction of PKG with GluA1-CT domain contributes to GluA1-S831 phosphorylation. A previous study shows that PKG regulates AMPA receptor functions by binding to PDZ motif which is downstream to the position of S831 in GluA1-CT domain (Snyder et al., 2000). In addition, activated PKA or PKG interacts with GluA1 directly or with anchoring proteins seems to regulate GluA1-S845 phosphorylation. PKA binds to A-kinase anchoring protein 79 and synapse-associated protein 97, a PDZ scaffold protein. This protein-protein complex causes the phosphorylation of S845 (Colledge and Scott, 1999). Activated PKG by cGMP increases its binding to GluA1 and this complex then phosphorylates S845 *in vivo* and *in vitro* (Serulle et al., 2007). These protein-protein interactions seem to play crucial roles in the GluA1 trafficking to the cell surface (Serulle et al., 2007; Schierberl et al., 2011). Together, these findings suggest that PKG regulates the phosphorylation of GluA1-S831 by interacting with a specific amino acid sequence called PDZ motif, which is apart from the S831 phosphorylation site. Consistent with this finding, CaMKII phosphorylates mGluR1 on threonine 871 which does not overlap with its binding site in the receptor (Jin et al., 2013). Binding and phosphorylation sites of extracellular signal-regulated kinase and c-Jun N-terminal kinase are not overlapped in the CT domain of mGluRs (Fantz et al., 2001; Remenyi et al., 2006; Zhou et al., 2006; Yang et al., 2017). Activation of protein kinases potentiates their interactions with GluA1 via an anchoring protein or directly, which seems to be important for the recruitment of GluA1 from the intracellular pool to the cellular surface (Serulle et al., 2007; Schierberl et al., 2011). For instance, A-kinase anchoring protein 150 (AKAP150) binds to PKA in the NAc. This protein-protein complex phosphorylates GluA1-S845 which regulates cocaine seeking behavior via dopamine D1 receptors (Guercio et al., 2018). Taken together, these data suggest that PKG binding to PDZ motif in GluA1-CT domain after repeated cocaine administration is necessary for phosphorylating GluA1-S831 which does not belong to the binding motif. This notion can be supported by the present finding that blockade of PKG binding to PDZ motif following repeated cocaine administration significantly reduced GluA1-S831 phosphorylation in the NAc.

Blockade of PKG binding to PDZ by intra-NAc infusion of interfering peptides reduced locomotor activity which was elevated after repeated cocaine administration in this study. This finding suggests that phosphorylation of S831 in GluA1-CT after repeated cocaine administration as a result of increasing PKG binding activity regulates behavioral changes. Previous studies show that interfering of a kinase binding to a receptor is found to prevent phosphorylation of the receptor, which alters the receptor functions (Medler and Bruch, 1999; Schaffhauser et al., 2000; Cai et al., 2001; Liu et al., 2009). Inhibition of PKG in the rat dorsal striatum and NAc decreases the repeated cocaine-induced increase in locomotor activity (Lee et al., 2013; Seo et al., 2013). Blockade of AMPA receptors in the rat NAc also decreases the repeated cocaine-induced increase in conditioned place preference (Zheng et al., 2015). Mutation of PKA binding site in AKAP150 reduces PKA-mediated S845 phosphorylation and AMPA receptor currents in the NAc shell (Guercio et al., 2018). Together, these data suggest that increased binding activity of PKG to PDZ motif after repeated cocaine administration seems to be required for GluA1-S831 phosphorylation, which may regulate behavioral changes. Recently, we have demonstrated that activation of PKG can regulate the phosphorylation of GluA1-S845 after repeated cocaine administration in the NAc (Seo et al., 2013). The fact that PKG is required for the phosphorylation of the two serine residues, 831 and 845, on the GluA1-CT domain of AMPA receptors may have an advantage in regulating the receptor functions in the NAc and the receptor-associated behavior after repeated exposure to cocaine.

## Conclusion

Repeated cocaine administration increases AMPA receptor GluA1-S831 phosphorylation in the NAc via PKG activation. Increased binding of PKG to PDZ domain in the CT of AMPA receptors after repeated cocaine administration is necessary for GluA1-S831 phosphorylation. Altered functions of the receptors via interaction of PKG may upregulate behavioral changes. Therefore, activation of PKG may potentiate the recruitment of GluA1 to the cellular surface, which seems to be required for GluA1 phosphorylation at S831, contributing to behavioral changes.

## Author Contributions

ESC, JHY, SYS and DKL designed the research. JHY, SYS and JHO designed Tat-peptides. JHY, SYS, JK and YR conducted the research. JHY, SYS and ISR performed data analysis. JHY, SYS and ESC wrote the manuscript.

## Conflict of Interest Statement

The authors declare that the research was conducted in the absence of any commercial or financial relationships that could be construed as a potential conflict of interest.

## Abbreviations

aCSF: artificial cerebro-spinal fluid
AMPA: α-amino-3-hydroxy-5-methyl-4-isoxazolepropionic acid
AKAP150: A-kinase anchoring protein 150
CaMKII: Ca^2+^-calmodulin-dependent protein kinase II
cGMP: cyclic guanosine monophosphate
Co-IP: co-immunoprecipitation
CT: carboxyl-terminus
DMSO: dimethylsulfoxide
i.p.: intraperitoneal
NAc: nucleus accumbens
NeuN: neuron-specific nuclear protein
NO: nitric oxide
PBS: phosphate buffered saline
PKA: protein kinase A
PKC: protein kinase C
PKG: protein kinase G
S831: serine 831
S845: serine 845
Tat: YGRKKRRQRRR amino acids sequence
Tat-GluA1-c: Tat-tagged scrambled peptide
Tat-GluA1-i: Tat-tagged interfering peptide
TBST: tris-buffered saline and Tween-20.
