# Supplementary material for: Activation of Protein Kinase G After Repeated Cocaine Administration Is Necessary for the Phosphorylation of α-Amino-3-Hydroxy-5-Methyl-4-Isoxazolepropionic Acid Receptor GluA1 at Serine 831 in the Rat Nucleus Accumbens
Source: Front Mol Neurosci. 2018 Jul 30;11:263. doi: 10.3389/fnmol.2018.00263 (PMC6077228; doi:10.3389/fnmol.2018.00263)
Supplement: Supplementary file 1 [file Table_1.pdf]

Supplementary Material

Activation of protein kinase G after repeated cocaine administration is necessary for the phosphorylation of α-amino-3-hydroxy-5-methyl-4-isoxazolepropionic acid receptor GluA1 at serine 831 in the rat nucleus accumbens

Ju Hwan Yang[+,1], Su Yeon Seo[+,1,2], Jeong Hwan Oh[1,3], In Soo Ryu[1,4], Jieun Kim[1], Dong Kun Lee[5], Yeonhee Ryu[2], Eun Sang Choe[1,*]

[1]Department of Biological Sciences, Pusan National University, South Korea

[2]Korea Institute of Oriental Medicine, South Korea

[3]Institute of Fisheries Sciences, Pukyong National University, South Korea

[4]Substance Abuse Pharmacology Group, Korea Institute of Toxicology, South Korea

[5]Department of Physiology, School of Medicine and Institution of Health Sciences, Gyeongsang National University, South Korea

[+]Equal contributors

*** Correspondence:** Eun Sang Choe, eschoe@pusan.ac.kr

Supplementary Table S1. Rates of changes in the immunoreactivity of pGluA1-S831 in the NAc after repeated systemic injections of saline or cocaine at different time points

	Repeated saline	Repeated cocaine
Time (min)	Mean ± SEM	
5	100 ± 15.412	127.33 ± 39.443
10	100 ± 12.428	129.873 ± 6.131
30	100 ± 16.493	422.652 ± 52.047*
60	100 ± 23.862	264.855 ± 24.21*
120	100 ± 4.517	164.846 ± 15.488#
240	100 ± 5.722	121.777 ± 3.903#

*$p < 0.05$ vs. repeated saline group at the same time point; #$p < 0.05$ vs. repeated cocaine group at the 30 min time point.

Supplementary Table S2. Rates of changes in the immunoreactivity of pGluA1-S831 in the NAc after intra-NAc infusion of KT5823 or Rp-cGMP prior to the final injection of saline or cocaine

		Repeated saline	Repeated cocaine
Treatment		Mean ± SEM	
WB	Vehicle	100 ± 14.02	260.8 ± 22.053*
	KT5823	114.225 ± 3.383	123.575 ± 14.446#
	Rp-cGMP	113.2 ± 2.587	88.3 ± 14.849#
IHC	Vehicle	100 ± 3.095	193.1 ± 11.656*
	KT5823	111.525 ± 4.509	111.025 ± 8.742#
	Rp-cGMP	107.025 ± 6.716	113.225 ± 7.035#

*p < 0.05 vs. repeated saline vehicle group; #p < 0.05 vs. repeated cocaine vehicle group; WB, western immunoblotting; IHC, immunohistochemistry.

Supplementary Table S3. Rates of changes in number of co-localization of pGluA1-S831 with NeuN in the NAc after the intra-NAc infusion of KT5823 or Rp-cGMP prior to the final injection of saline or cocaine

	Repeated saline	Repeated cocaine
Treatment	Mean ± SEM	
Vehicle	100 ± 2.68	184.895 ± 3.815*
KT5823	99.248 ± 4.4	95.873 ± 4.09[#]
Rp-cGMP	106.62 ± 5.262	88.951 ± 4.784[#]

*$p < 0.05$ vs. repeated saline vehicle group; [#]$p < 0.05$ vs. repeated cocaine vehicle group.

Supplementary Table S4. Rates of changes in the immunoreactivity of PKG, pGluA1-S831 or GluA1 in the GluA1 precipitate of the NAc after the repeated systemic injections of saline or cocaine

	Repeated saline	Repeated cocaine
Treatment	Mean ± SEM	
PKG	100 ± 5.289	228.4 ± 13.16*
pGluA1^{S831}	100 ± 11.53	202 ± 9.809*
GluA1	100 ± 8.264	106.7 ± 8.209

*p < 0.05 vs. repeated saline group.

Supplementary Table S5. Rates of changes in the immunoreactivity of pGluA1-S831 after intra-NAc infusion of Tat-peptides prior to the final injection of saline or cocaine

	Repeated saline	Repeated cocaine
Treatment	Mean ± SEM	
Vehicle	100 ± 2.634	200.74 ± 2.837*
Tat-GluA1-c (0.2 nmol)	98.36 ± 2.837	181.64 ± 11.163*
Tat-GluA1-c (2.0 nmol)	95.48 ± 0.856	176.46 ± 10.206*
Tat-GluA1-i (0.2 nmol)	106.96 ± 4.599	175.62 ± 3.267*
Tat-GluA1-i (2.0 nmol)	82.3 ± 3.578	99.82 ± 6.098#

*$p < 0.05$ vs. repeated saline vehicle group; #$p < 0.05$ vs. repeated cocaine vehicle group.

Supplementary Table S6. Rates of changes in the immunoreactivity of PKG or GluA1 in the GluA1 precipitate after the intra-NAc infusion of Tat-peptides prior to the final injection of saline or cocaine

		Repeated saline	Repeated cocaine
Treatment		Mean ± SEM	
PKG	Vehicle	100 ± 4.293	214.933 ± 7.031*
	Tat-GluA1-c (2.0 nmol)	107.667 ± 8.019	169.267 ± 13.762*
	Tat-GluA1-i (2.0 nmol)	112.933 ± 12.203	93.833 ± 6.912#
GluA1	Vehicle	100 ± 1.02	97 ± 0.603
	Tat-GluA1-c (2.0 nmol)	98.1 ± 2.854	95.233 ± 6.306
	Tat-GluA1-i (2.0 nmol)	100.8 ± 0.874	95.1 ± 4.912

*$p < 0.05$ vs. repeated saline vehicle group; #$p < 0.05$ vs. repeated cocaine vehicle group.

Supplementary Table S7. Changes in total distance travelled for 60 min after the intra-NAc infusion of Tat-peptides prior to the final injection of saline or cocaine

	Repeated saline	Repeated cocaine
Treatment	Mean ± SEM	
Vehicle	1068.9 ± 144.882	17617.2 ± 1574.275*
Tat-GluA1-c	1093.5 ± 83.314	15075.6 ± 1531.533*
Tat-GluA1-i	863.6 ± 126.171	5649.4 ± 379.234#

*$p < 0.05$ vs. repeated saline vehicle group; #$p < 0.05$ vs. repeated cocaine vehicle group.
